# Real-time decoding of full-spectrum Chinese using brain-computer interface

**DOI:** 10.1126/sciadv.adz9968

**Published:** 2025-11-05

**Authors:** Youkun Qian, Changjiang Liu, Peixi Yu, Xingchen Ran, Shurui Li, Qinrong Yang, Yang Liu, Lei Xia, Yijie Wang, Jianxuan Qi, Erda Zhou, Junfeng Lu, Yuanning Li, Tiger H. Tao, Zhitao Zhou, Jinsong Wu

**Affiliations:** ^1^ Department of Neurosurgery, Huashan Hospital, Shanghai Medical College, Fudan University, Shanghai 200040, China.; ^2^ Shanghai Key Laboratory of Clinical and Translational Brain-Computer Interface Research, Shanghai 200040, China.; ^3^ National Center for Neurological Disorders, Huashan Hospital, Shanghai Medical College, Fudan University, Shanghai 200040, China.; ^4^ 2020 X-Lab, Shanghai Institute of Microsystem and Information Technology, Chinese Academy of Sciences, Shanghai 200050, China.; ^5^ School of Biomedical Engineering, ShanghaiTech University, Shanghai 201210, China.; ^6^ State Key Laboratory of Advanced Medical Materials and Devices, ShanghaiTech University, Shanghai 201210, China.; ^7^ Neuroxess Co. Ltd., Shanghai 200023, China.; ^8^ School of Graduate Study, University of Chinese Academy of Sciences, Beijing 100049, China.; ^9^ MOE Frontiers Center for Brain Science, Huashan Hospital, Fudan University, Shanghai 200040, China.; ^10^ Shanghai Clinical Research and Trial Center, Shanghai 201210, China.; ^11^ Lingang Laboratory, Shanghai 200031, China.; ^12^ Guangdong Institute of Intelligence Science and Technology, Hengqin, Zhuhai, Guangdong 519031, China.; ^13^ Tianqiao and Chrissy Chen Institute for Translational Research, Shanghai, China.; ^14^ State Key Laboratory of Transducer Technology, Shanghai Institute of Microsystem and Information Technology, Chinese Academy of Sciences, Shanghai 200050, China.; ^15^ School of Integrated Circuits, University of Chinese Academy of Sciences, Beijing 100049, China.

## Abstract

Speech brain-computer interfaces (BCIs) offer a promising means to provide functional communication capacity for patients with anarthria caused by neurological conditions such as amyotrophic lateral sclerosis (ALS) or brainstem stroke. Current speech decoding research has predominantly focused on English using phoneme-driven architectures, whereas real-time decoding of tonal monosyllabic languages such as Mandarin Chinese remains a major challenge. This study demonstrates a real-time Mandarin speech BCI that decodes monosyllabic units directly from neural signals. Using the 256-channel microelectrocorticographic BCI, we achieved robust decoding of a comprehensive set of 394 distinct syllables based purely on neural signals, yielding median syllable identification accuracy of 71.2% in a single-character reading task. Leveraging this high-performing syllable decoder, we further demonstrated real-time sentence decoding. Our findings demonstrate the efficacy of a tonally integrated, direct syllable neural decoding approach for Mandarin Chinese, paving the way for full-coverage systems in tonal monosyllabic languages.

## Teaser

Real-time decoding of full-spectrum Chinese syllables using flexible high-density ECoG achieves high decoding accuracy.

## INTRODUCTION

Human language constitutes an intrinsic higher-order neurocognitive faculty, serving as a defining feature of human cognition. Neurological disorders such as stroke, brain tumors, or neurodegenerative diseases can severely impair speech and language function, profoundly affecting communication and quality of life for patients and their families (*1*, *2*). Brain-computer interfaces (BCIs) that decode speech directly from neural signals offer a promising avenue to restore communication in such individuals (*3*, *4*). Although speech production relies on a distributed cortical network, decoding intended speech from neural activity—particularly at the level of internal speech or abstract thought—remains a major challenge (*5*, *6*). A leading approach focuses on the ventral sensorimotor cortex (vSMC), which encodes articulatory kinematic trajectories (*7*). Neural signals from this region can be transformed into discrete linguistic units or articulatory gesture parameters and subsequently synthesized into words, sentences, or sounds (*8*–*10*). This strategy is especially suitable for individuals with intact speech motor areas, aiming to re-enable functional communication. Recent advances in English language decoding have enabled real-time translation of brain activity into text or speech for patients with severe dysarthria caused by conditions such as amyotrophic lateral sclerosis (ALS) or brainstem stroke (*11*–*15*).

Nevertheless, achieving reliable decoding in tonal languages—especially Mandarin Chinese, where pitch variations convey lexical meaning—remains a critical unmet challenge in the field (*16*). Unlike languages such as English, where multiphonemic redundancy enables error compensation through statistical language models (*17*), Mandarin Chinese is characterized by monosyllabic morphemes, lexical tones that carry semantic significance, and exceptionally high homophone density (*18*, *19*). Mandarin’s minimal phonetic redundancy amplifies error risks: A single phonemic deviation can generate a lexically valid but contextually incongruent syllable. This structural constraint imposes stringent demands on decoding precision, particularly for clinical BCIs requiring near-perfect accuracy.

In this study, we propose the Mandarin syllable—encompassing both segmental and tonal information—as an optimal intermediate decoding unit. Syllables offer a more stable representation than phonemes and are linguistically meaningful, making them more robust against minor decoding errors. We present a real-time BCI framework that decodes the full spectrum of spoken Mandarin syllables from high-density electrocorticography (ECoG) recordings collected from a participant undergoing clinical epilepsy monitoring. We achieved an offline syllable-level decoding accuracy of 71.2% using neural signals alone. Building on this, we implemented a hierarchical character-to-sentence decoding strategy, the final system enabled communication at a rate of 49.7 characters/min (CPM). These findings demonstrate the feasibility of syllable-based decoding for Mandarin and provide a scalable strategy for real-time speech neuroprosthesis in tonal languages with monosyllabic morphemes across diverse linguistic populations (*20*).

## RESULTS

### Overview of the experimental tasks and decoding pipeline

**Fig. 1.**
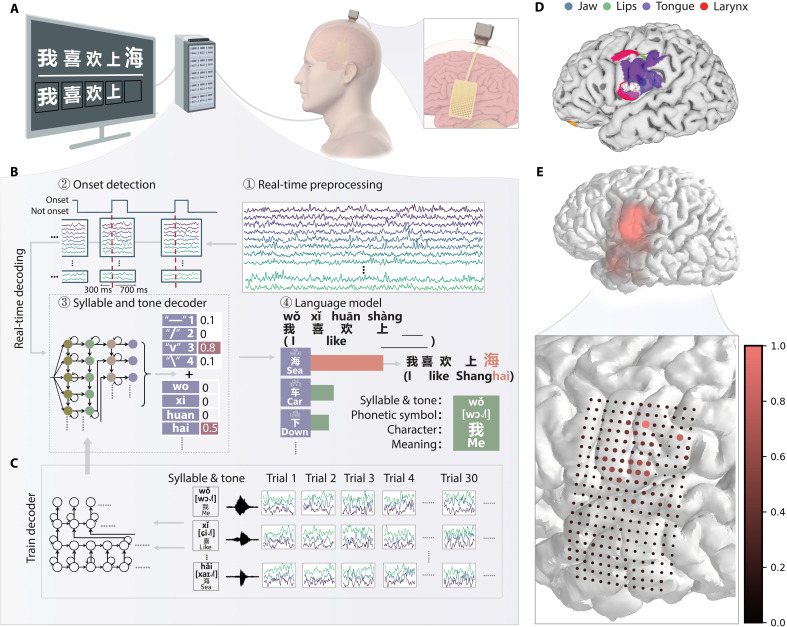
Framework of a real-time BCI for decoding Chinese sentences. (**A**) Schematic overview showing the participant implanted with a flexible 256-channel ECoG array performing the real-time sentence decoding task. (**B**) Real-time decoding pipeline: ① Raw ECoG signals were preprocessed in overlapping 50-ms windows (10-ms step) to extract high-γ activity. ② A dedicated model detects speech onset and align a 1000-ms neural segment centered on the onset (−300 to +700 ms) for decoding. ③ Data are then fed into a dual-stream decoder to compute probability distributions over syllables and tones. ④ The resulting syllable-tone combinations are matched to candidate Chinese characters, and a language model outputs the most likely prediction by integrating neural decoding probabilities with linguistic priors. The green square is an example to illustrates the character-level information, including syllable, tone, phonetic symbol, character, and semantic meaning. (**C**) The syllable and tone decoder were trained using data from 394 distinct syllables, each repeated multiple times across trials. (**D**) Preoperative fMRI was used to localize the oromotor cortex (adjusted *P* value < 0.05, cluster-based correction). (**E**) Electrodes cumulatively contributing to 90% of decoding performance were visualized using cortical heatmaps (Gaussian smoothing), with individual contributions represented by dot size and color intensity.

In this study, we recorded neural signals from a 43-year-old right-handed female participant who implanted a high-density flexible ECoG array as part of her clinical treatment for seizure localization. On the basis of clinical treatments needs, the 256-channel flexible ECoG array was placed over the area of the middle temporal gyrus, superior temporal gyrus, vSMC, and small part of the pars opercularis. Considering the specialty of Chinese language, the participant performed two tasks: (i) single-character reading task, in which individual Chinese characters were presented on the screen and the participant read each character aloud three times. For characters that the participant does not recognize, the corresponding voice will be played synchronously when the word appears to assist reading; (ii) sentence-reading task, in which the participant read a whole sentence on the screen (Fig. 1A). The first task served to train a full-spectrum neural decoder, whereas the second task was designed to evaluate its effectiveness in real-time sentence decoding.

To implement this real-time decoding system, we designed a multistage pipeline that transforms raw neural signals into linguistic outputs in a continuous and low-latency manner. Specifically, high-γ neural activity (70 to 170 Hz) was extracted and processed in 50-ms sliding windows stepped every 10 ms (*9*, *21*, *22*). An onset event was identified on the basis of the aligned signal, and once speech was detected, the relevant signal segment drove a dual-stream decoder (*23*), yielding concurrent probability distributions over Mandarin syllables and tones. The resulting probabilities for each tonal syllable were then mapped to a corresponding set of candidate characters. Last, a beam search algorithm processed these candidate characters. It searched for the optimal sequence by simultaneously considering the likelihood on the basis of neural evidence and the probability from a 3-gram language model. The language model was trained using a Mandarin corpus and included both commonly used daily expressions and command-style phrases applicable to future use cases such as robotic arm or avatar control. The character yielding the highest combined score was determined as the final output character (Fig. 1B).

The syllable and tone decoders were initially trained on data from the single-character reading task, based on the working hypothesis that the neural representation of syllables shares some features between isolated character and sentence production. These models were then fine-tuned using the sentence task and applied for real-time sentence decoding (Fig. 1C).

Preoperative functional magnetic resonance imaging (fMRI) shows that there were high signals related to articulatory movement (jaw, lips, tongue, and larynx) in the vSMC (Fig. 1D). To investigate the relationship between fMRI-identified activation areas and electrode importance for decoding, we quantified the contribution of each electrode in the ECoG array. Contribution maps and heatmaps revealed substantial spatial correspondence between fMRI activation patterns and decoding contributions, demonstrating that electrodes overlying the functionally localized oromotor cortex (representing jaw, lip, tongue, and pharynx movements) exhibited high importance for decoding performance (Fig. 1E).

### Rationale for syllable-based stimulus design and decoding strategy

**Fig. 2.**
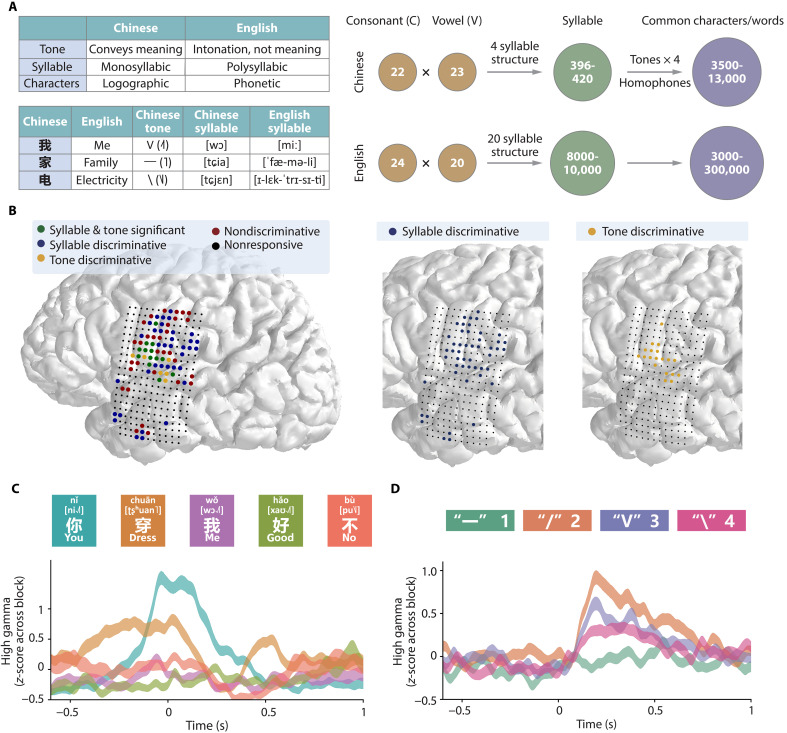
Uniqueness of Chinese and cortical electrodes distinguishing Chinese syllables and tones. (**A**) The upper table illustrates the differences between Chinese and English. In Chinese, tone conveys meaning, and characters are monosyllabic and logographic. The number of vowels and consonants in both languages is similar, yet Chinese has fewer syllable structures, so fewer syllables compared to English. Specifically, Chinese contains approximately 418 syllables, which form around 3500 commonly used characters and approximately 13,000 modern characters after considering homophones and four tones. (**B**) Categorization of electrodes based on their differential responsiveness for Chinese syllables and tones. Blue electrodes are discriminative for syllables, identified using a spatiotemporal cluster-based permutation test (cluster-level *P* < 0.05) followed by pairwise Welch’s *t* tests (*P* < 0.05, Bonferroni corrected) to ensure differentiation among at least three syllables. Yellow electrodes are discriminative for tones, identified by a one-way ANOVA, revealing significant high-γ signal differences among the four tones (*P* < 0.05, Bonferroni corrected). (**C** and **D**) High-γ signal for electrode101 across five syllables and electrode124 across four tones. Time 0 indicates the sound onset, and mean high-γ activities represent averages across 90 trials.

The decoding strategy adopted in this study was fundamentally shaped by the unique phonological and structural features of Mandarin Chinese. Mandarin is tonal, predominantly monosyllabic, and characterized by a simple syllable structure lacking complex consonant clusters (*19*, *24*). Although both tonal and nontonal languages use the dorsal and ventral laryngeal motor cortex (LMC) to modulate vocal pitch, tonal languages exhibit more precise bidirectional pitch control, encompassing both rising and lowering commands to define lexical meaning (*25*, *26*). As a logographic language, each character carries distinct form, pronunciation, tone, and meaning. For example, the character “我” (meaning “me,” pinyin “wǒ”) is pronounced [wɔ] with a third tone (V, IPA symbol [˨˩˦]). Mandarin contains a full spectrum of approximately 418 base syllables, which expand to 1298 tonal syllables when considering the four lexical tones. These syllables correspond to 3500 commonly used characters and approximately 13,000 characters in total (*19*) (Fig. 2A). This results in a high prevalence of homophones (e.g., the tonal syllable [t͡ɕia˥] can correspond to over 20 different characters, such as “家” family, “加” plus, and “佳” good). Consequently, directly decoding such a vast number of individual characters from neural signals remains impractical.

Instead, a more tractable approach is to decode phonological units, particularly full tonal syllables, from brain activity. If phonemes were used as decoding units—as is common in English BCI systems—the phonemic conciseness of Mandarin characters (typically one to four phonemes) implies that even a single phoneme decoding error can readily transform the sequence into the valid phonemic representation of an entirely different syllable and thus a different character. In such cases, language models would have limited ability to perform effective error correction or disambiguation, ultimately resulting in reduced accuracy.

We therefore propose decoding full tonal syllables as a more robust and linguistically appropriate strategy. Syllables are more stable, semantically informative units than phonemes, and their total number remains within a manageable range for direct pattern recognition from neural signals.

To enable this, we designed a single-character reading task in which participants repeatedly read individual Chinese characters representing target tonal syllables. An initial set of 418 unique syllables was selected to cover the full spectrum of Mandarin syllables from the *Modern Chinese Dictionary* and was refined to 394 syllables on the basis of participant familiarity and inclusion of two idiosyncratic pronunciation (“king” and “len”) (table S1). This meticulous stimulus selection and task design aimed to ensure balanced exposure and promote clear articulation for each target syllable. To further evaluate decoding performance in real-world speech and assess the contribution of the language model, we developed a sentence-reading task. Sentences ranged from 2 to 11 characters and were read word by word with deliberate pauses. This allowed us to fine-tune the syllable-based decoders and test their generalizability.

### Data collection and neural correlates of syllable production

Over 11 days of intracranial ECoG monitoring, ~9 hours of neural data were collected as the participant performed the character-reading task with the 394 selected syllables. The recording schedule involved varying repetitions: 130 syllables were read 60 times each (days 3 to 7), and 264 syllables were read 30 times each (days 8 to 11) (figs. S2 and S3). Some syllables were presented for additional repetitions beyond these baseline counts.

Throughout the recording period, the implanted ECoG electrodes generally performed reliably, maintaining a high signal-to-noise ratio (SNR), exhibiting minimal signal drift across days, and showing no significant emergence of new bad channels over time (figs. S4 and S5). Analysis of these ECoG signals revealed distinct neural correlates for syllable and tone processing. Electrodes demonstrating significant discriminative capacity for both syllables and tones were predominantly localized to the vSMC (Fig. 2B), consistent with the prior literature (*9*, *26*, *27*). Within syllable-discriminative electrode sites in the vSMC, distinct mean high-γ activities were observed for different syllables, indicating unique neural signatures (Fig. 2C, example mean high-γ activities for different characters like 你(you), 穿(dress), 我(me), 好(good), and 不(no), each represent a unique syllable). Notably, these vSMC sites also exhibited distinct high-γ responses to varying articulatory manners and places of the initial consonants within these syllables (fig. S6), suggesting an articulatory basis for the observed syllable-level mean high-γ activities differences. Although tone-discriminative electrodes were also identified in the vSMC, their activity was less prominent than expected (Fig. 2, B and D), potentially due to electrode coverage being limited to the inferior aspects of the vSMC and not fully encompassing the LMC (*26*). In addition, task-related activity was noted in the middle temporal gyrus, possibly reflecting processing of self-listening and Chinese-specific orthographic-phonological mappings (*28*).

### Decoder architecture and performance on offline syllable and tone decoding

**Fig. 3.**
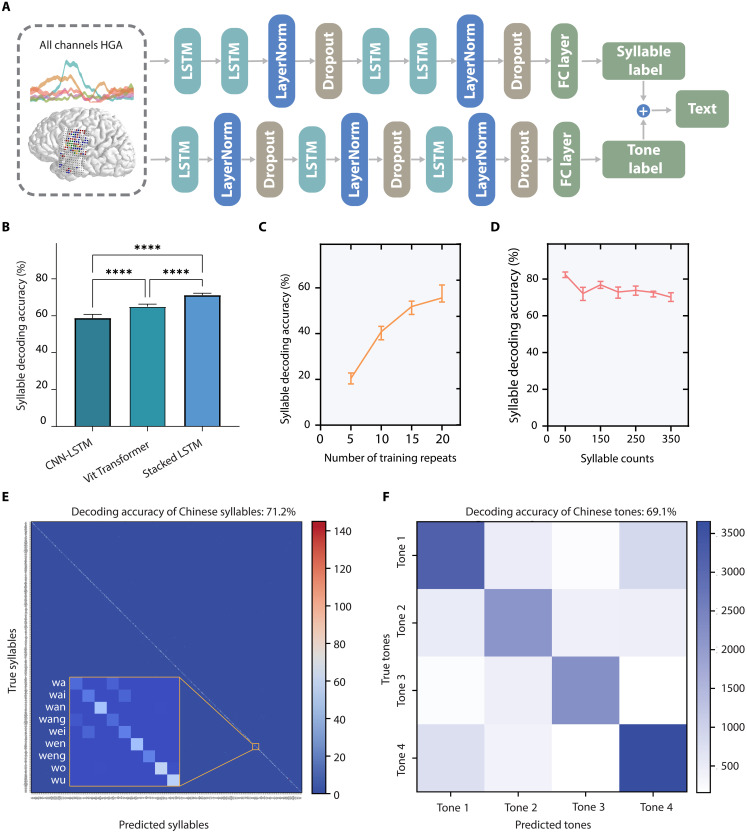
Offline performance of syllable and tone decoding. (**A**) Architecture of the dual-stream decoder. A 1000-ms segment of high-γ neural activity from all channels, centered on speech onset, is fed into two parallel four-layer LSTM networks to decode the syllable and tone, respectively. The outputs are then combined and translated into corresponding Chinese characters. (**B**) Comparison of the median decoding accuracy for 394 syllables across three models (CNN-LSTM, ViT, and Stacked LSTM), evaluated with 10-fold cross-validation. ****P < 0.0001 (one-way ANOVA with Tukey’s post-hoc test). (**C**) Syllable decoding accuracy as a function of the number of training repetitions per syllable. (**D**) Syllable decoding accuracy as a function of the total number of unique syllables included in the decoding set. For (B) to (D), error bars represent the 99% CI calculated via bootstrap procedure with 1000 resamples. (**E**) Confusion matrix for decoding 394 Chinese syllables (see detail in fig. S9). The inset details confusion among syllables that start with the initial “w.” (**F**) Confusion matrix for decoding the four Mandarin Chinese tones. For (E) and (F), heatmap colors indicate the number of correctly decoded trials.

Informed by prior works and the linguistic structure of Mandarin, we developed a dual-stream decoding architecture that processes syllable and tone information in parallel before integrating these streams for character generation (Fig. 3A). For the syllable and tone decoding, we evaluated several neural network architectures, including a Convolutional Neural Network (CNN)–Long Short-Term Memory (LSTM), a Vision Transformer–based model (ViT), and a custom four-layer LSTM network (hyperparameters in tables S3 to S5). All architectures demonstrated decoding accuracies significantly above the chance level of 0.25% (*P* < 0.0001 for all three methods, one-sample *t* tests). Comparative analysis revealed that the four-layer LSTM architecture achieved the highest syllable decoding accuracy (Fig. 3B), with a median accuracy of 71.2% (99% confidence interval (CI): [70.1, 72.2]) on the full-spectrum 394 syllable set through 10-fold cross-validation, superior to both the CNN-LSTM and ViT [*P* < 0.0001 for both pairwise comparisons, one-way analysis of variance (ANOVA) with Tukey’s post hoc tests]. The confusion matrix for syllable decoding illustrates generally high discriminability across syllable types, with some expected confusability between phonetically similar syllables (Fig. 3E and fig. S9). Nonetheless, the model successfully distinguished syllables differing only by their vowel even when sharing the same initial consonant.

To understand the data requirements for robust decoding, we investigated the impact of training data volume on performance. Syllable decoding accuracy showed a clear dependence on the number of training repetitions per syllable, rising from 20.4% (99% CI: [18.0, 22.8]) with five repetitions to 55.6% (99% CI: [53.8, 61.2]) with 20 repetitions (Fig. 3C). Furthermore, we assessed the scalability of our approach by examining performance as a function of the number of unique syllables included in the training set. Decoding accuracy remained relatively stable, exhibiting only a marginal decline as the syllable set size increased from 50 to 350 unique syllables, suggesting the model’s capacity to handle a large vocabulary (Fig. 3D). Last, despite electrode coverage not explicitly targeting the LMC in this participant, tone decoding median accuracy was 69.1% (99% CI: [66.0, 71.6]), significantly above chance level 25% (Fig. 3F, tone confusion matrix). This indicates that sufficient tone-related information could be extracted from the implanted vSMC and surrounding areas.

### Sentence decoding performance and BCI system control

**Fig. 4.**
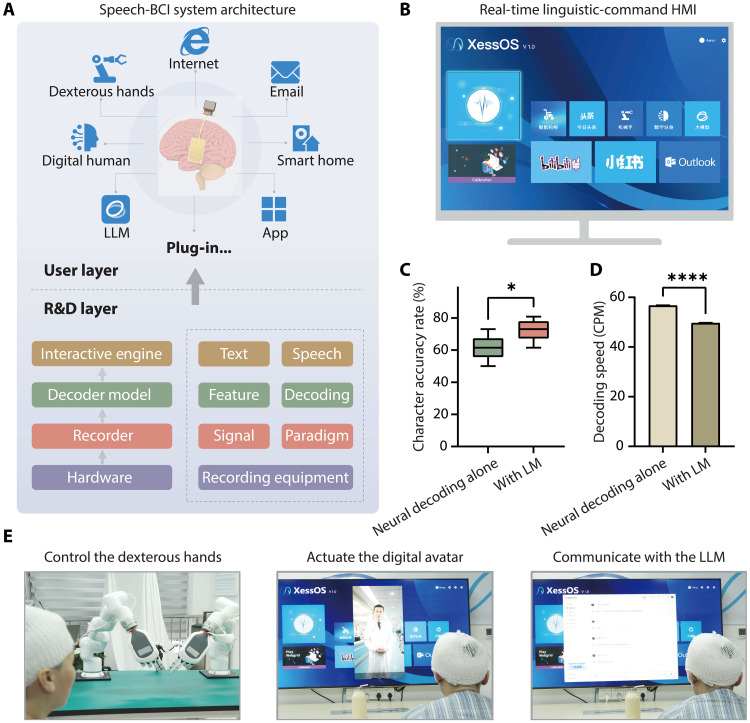
Real-time speech decoding and speech-based BCI for controlling multiple software and hardware systems. (**A**) System architecture of the speech-based BCI capable of operating multiple plug-ins. Brain signals are decoded into text or speech to control various software and hardware systems such as dexterous robotic hands, digital avatars, and large language models (LLMs). The system also enables functions including internet browsing, email sending, smart-home control, and other applications. (**B**) Interface of the real-time linguistic-command human-machine interaction (HMI) system. (**C**) Real-time decoding accuracy of a limited sentence set with and without a language model (LM). Box plots represent the median accuracy (central line), 25th and 75th percentiles (box), and the minimum to maximum range (whiskers). *P < 0.05 (paired *t* test). (**D**) Comparison of the decoding speed with and without an LM, calculated from the same sentence dataset. Error bars indicate the range from minimum to maximum values. ****P < 0.0001 (paired *t* test). (**E**) Real-world applications of the speech-based BCI demonstrating control of dexterous robotic hands, activation of digital avatars, and interactive communication with an LLM.

The collection of data across 394 unique Mandarin tonal syllables in this study establishes a comprehensive foundation for advancing toward open-vocabulary speech neuroprosthesis. To evaluate sentence-level decoding capabilities, the core decoder model was first trained on all single-character reading task collected from the initial 10 days of recording. This base model was then fine-tuned using all available sentence-reading data also acquired within these first 10 recording days. Real-time sentence decoding performance was subsequently assessed using sentences presented in the final recording session on day 11. Because of time constraints, speech onset on this day was detected using the audio signal rather than neural activity (see Materials and Methods). Our real-time decoding system yielded a character accuracy rate (CAR) of 61.5% (99% CI: [50.0, 73.1]) on the basis of direct neural decoding alone. With the integration of a 3-gram language model, the CAR significantly improved to 73.1% (99% CI: [61.5, 80.8]) (Fig. 4C and fig. S10). The corresponding communication rate achieved was 56.7 (99% CI: [56.5, 56.8]) CPM from neural decoding and 49.7 (99% CI: [49.5, 49.7]) CPM when the language model was incorporated (Fig. 4D). To demonstrate the practical utility of this real-time decoding, we developed and implemented an integrated BCI system. This architecture facilitated the connection of decoded speech intentions to various external applications (Fig. 4A). A key component of this system was a user interface, designed to allow the participant to select desired functions from a predefined set (Fig. 4B). The participant successfully used her decoded speech to control a robotic arm, to generate vocalizations through a digital avatar, and to interact with a large language model (Fig. 4E). In these proof-of-concept demonstrations, the decoding system achieved a CAR of 78.3% for robotic arm control, with 54.0% command accuracy (one to three characters per command; full match required), 76.9% for digital avatar, and 65.4% for language model interaction.

## DISCUSSION

Previous ECoG-based investigations have explored the neural mechanism of Mandarin tone production (*26*) and achieved offline decoding of limited sets of syllables and sentences (*29*, *30*). Although studies using stereoelectroencephalography (SEEG) have demonstrated broader syllable-level decoding and partial sentence reconstruction, achieving high neural decoding accuracy remains a major challenge (*31*, *32*).

Building on these earlier efforts, our study demonstrates that combining high-density, ultraconformal ECoG grids with a syllable-centric decoding framework can yield substantial improvements. The ECoG arrays provided broad and stable cortical coverage, particularly over speech-related regions, and enabled us to decode a large set of 394 Mandarin tonal syllables with high accuracy—based primarily on neural features before any linguistic postprocessing. This robust syllable-level decoding proved to be a critical foundation for our subsequent success in real-time sentence decoding.

However, one current limitation is that, because of time constraints, we did not fully integrate neural-based onset detection into the real-time decoding system for this participant. Instead, speech onset was identified using the audio signal. To address this, we subsequently developed a neural-based real-time onset detection method. This method, tested using both this participant’s data and data from an additional epilepsy patient, remained reliable even during covert articulation, thus supporting its broader generalizability beyond overt speech tasks (fig. S7).

A major challenge of the present study is its reliance on a single participant, which constrains the generalizability of our findings. Future work will therefore focus on multisubject validation to establish the robustness and stability of the real-time decoding framework. To improve cross-participant generalizability, one promising direction is to construct a foundational model trained on data aggregated from multiple participants. Individual electrode locations could be mapped onto a standardized brain atlas (e.g., MNI152 space) or atlas-based parcellations (e.g., Human Connectome Project multimodal parcellation) using preoperative fMRI (*33*, *34*). By incorporating anatomical information as parameters within the decoding algorithm, it may allow the model to learn a more fundamental neural representations shared across individuals. When applied to a new participant, transfer learning could then be performed by fine-tuning with participant-specific data. This approach has the potential to shorten training time and enhance decoding accuracy.

Several directions could further advance this technology and enhance its clinical applicability. First, integrating ECoG with complementary biosignals—such as facial images, magnetoencephalography (MEG), or electromyography (EMG) from residual facial or laryngeal muscles—may substantially improve the system’s robustness, adaptability, and accuracy, especially under naturalistic conditions (*35*). Second, developing wireless, fully implantable ECoG systems aims to reduce infection risks, prolong implantation duration, and thereby expand longitudinal data collection across individuals. Third, applying these implants to patients with severe speech disorders (e.g., ALS and stroke) captures cross-patient neural variability, enabling generalizable Chinese language decoding models.

Beyond improvements in decoding accuracy and hardware performance, expanding the neural targets of speech BCIs represents an exciting frontier. Although current approaches primarily leverage signals from motor and premotor cortices responsible for articulation, future systems may benefit from incorporating activity in higher-order language (*35*–*39*), such as the middle temporal gyrus, inferior frontal gyrus, and supramarginal gyrus. Integrating the semantic and syntactic information processed within these regions may help build more stable and accurate speech decoders. This could also extend BCI applications to patients with extensive cortical damage, such as those with large territory strokes, thereby enabling restoration of broader language functions and not merely speech production.

## MATERIALS AND METHODS

### Participant

A 43-year-old female patient undergoing presurgical evaluation for epilepsy at Huashan Hospital, Fudan University (Shanghai, China) participated in this study. Following assessment by a senior neurologist, a neural signal acquisition system was surgically implanted, with the flexible high-density ECoG arrays placed on the cortical surface and the connected headstage securely fixed to the skull by an experienced neurosurgeon (fig. S1B). This initial procedure was conducted primarily to localize the epileptogenic focus in preparation for therapeutic resection. All research procedures involving the participant were approved by the Institutional Review Board of Huashan Hospital, Fudan University (approval no. KY2024-842) and were conducted in accordance with institutional and ethical guidelines. The participant was fully informed of the study procedures and the voluntary nature of her participation and that all collected data would be used exclusively for research purposes. Written informed consent was obtained before the initiation of any research-related activities. The ECoG arrays remained implanted for a total of 13 days, during which the participant underwent continuous clinical monitoring alongside intermittent research data collection sessions. After this period, the electrodes were removed, and the identified epileptogenic focus was surgically resected in a second planned procedure as part of her clinical treatment.

### Integrated ECoG implant system

The integrated implant (Neuroxess Co. Ltd.) comprises four main components (fig. S1A): a flexible high-density ECoG electrode array, a flexible printed circuit (FPC), a signal processing unit, and a titanium enclosure. The core component is the ECoG electrode array, which has 256 electrodes, with each recording contact having a diameter of 1.3 mm and a center-to-center interelectrode distance of 3 mm.

The electrode array is bonded to the FPC to facilitate signal transmission and allow simultaneous addressing and acquisition of 256 electrode channels. The signal processing unit uses four Intan RHD2164 chips, which effectively suppress noise and amplify signals while maintaining low power consumption. Last, a customized titanium enclosure serves as the encapsulation for the integrated implant, ensuring excellent waterproofing, outgassing resistance, and biocompatibility. This design guarantees the stable performance of the ECoG BCI during long-term operation.

### Experimental design

In Mandarin Chinese, each character corresponds to a phonetic representation known as “pinyin,” which indicates its pronunciation. However, because of the participant’s limited educational background, she was unable to read pinyin directly. Instead, to establish a comprehensive syllable set, we listed all 418 Mandarin syllables (table S1) and selected several common characters for each. For each target syllable, one character that she could confidently read and pronounce was selected as the experimental stimulus. When the participant could not recognize any character for a given syllable, an audio prompt was provided to assist.

In the character-reading task, individual Chinese characters were presented visually on the screen (fig. S2A). The participant was instructed to read each character three times, with self-paced intervals between repetitions (mean interval: 1.38 ± 0.53 s; average reading speed: 30.45 ± 4.60 CPM). After one character was read three times, the researcher manually advanced to the next character. For syllables where the participant was unfamiliar with all corresponding characters, an audio recording of the correct pronunciation was played before she attempted to repeat the syllable three times. In the sentence-reading task, full sentences were presented on the screen, and the participant read them aloud at a self-determined pace similar to the single-character task. Throughout all reading tasks, an automatic speech recognition model monitored the participant’s pronunciation in real time. If the pronunciation was correct, the character turned green on the screen to provide immediate visual feedback. In the real-time sentence decoding task, a fixed set of five sentences was read by the participant at a pace similar to the preceding tasks. Each sentence was repeated once per trial, across six trials. Together, the reading tasks covered 394 distinct Mandarin syllables (see table S1).

### Preoperative fMRI analysis

Participants performed a block-design language localizer task (on: 4 s, off: 8 s) with four motor conditions: jaw opening, tongue curling, lip protrusion, and vocalizing “yi” (larynx), each repeated 10 times. fMRI images were acquired on a 3T United Imaging uMR790 scanner (repetition time = 2 s, voxel size = 2.5 mm^3^, T2-weighted echo-planar imaging). Preprocessing included slice timing correction, distortion correction, motion correction, and alignment to the T1-weighted structural image. Four motion regions of interest were defined on the basis of the general linear model (GLM) contrast analysis and visualized on the cortical surface.

### Data preprocessing

Synchronized ECoG and audio signals were acquired at a sampling rate of 15 kHz. Distinct ECoG processing pipelines were used for offline analysis and real-time decoding, following established methodologies for ECoG signal processing and feature extraction (*9*, *21*, *22*).

For offline processing, the raw 15-kHz ECoG signals were preprocessed through the following steps: (i) rejection of bad channels; (ii) common average referencing; (iii) downsampling to 400 Hz; (iv) removal of power-line noise (50 Hz) and its harmonics using zero-phase IIR notch filters; (v) band-pass filtering of high-γ activity (70 to 150 Hz) using a Gaussian filter; (vi) extraction of the analytic amplitude via the Hilbert transform; and (vii) *z*-score normalization across time for each channel. This offline-processed data were used to identify syllable, tone or onset discriminative electrodes, visualize mean high-γ activity, and generate t-distributed Stochastic Neighbor Embedding (t-SNE) embeddings for dimensionality reduction (Fig. 2 and figs. S5 and S6).

For real-time decoding, ECoG signals were streamed in continuous 10-ms segments. The streaming data underwent the following steps: (i) rejection of bad channels; (ii) common average referencing; (iii) removal of power-line noise (50 Hz) and its harmonics using IIR notch filters; (iv) band-pass filtering between 70 to 170 Hz using a third-order Butterworth filter; (v) computing the analytic envelope via the Hilbert transform. High-γ activity (HGA) features for each channel and time point were calculated by averaging the analytic envelope within a 50-ms sliding window (a step size of 10 ms); and (vi) *z*-scoring using fixed normalization parameters, where the mean and SD were computed from all character-reading task collected before the final day. All filters were causal and maintained internal state. This real-time processing pipeline was used both to generate features for decoder training and to perform online decoding during the experimental sessions (Figs. 3 and 4 and figs. S9 and 10).

### SNR calculation

To evaluate ECoG signal quality, the SNR was computed for each channel. Raw 15-kHz signals, after initial 50 Hz (and harmonics) notch filtering, were further band-pass filtered between 1.5 Hz (high-pass) and 250 Hz (low-pass). Segments containing overt artifacts were manually rejected. The resulting data were processed in 60-s windows with a 30-s step. Within each 60-s window, the Hilbert envelope was calculated and then averaged into 100-ms bins. These bins were subsequently classified as “high activity” or “low activity” on the basis of their mean envelope amplitude relative to the window’s overall activity. The “signal” component was defined as the peak-to-peak (*PP*) amplitude within contiguous periods of high activity (minimum duration of 400 ms, i.e., four consecutive high-activity bins). The “noise” component was the root mean square (*RMS*) value calculated from periods identified as low activity. The *SNR* was then determined using the formula (*40*)

$$\text{SNR}=20*\text{lo}g_{10}\left[\frac{\frac{1}{N_{\text{High}}}\sum_{n=1}^{n=N_{\text{High}}}\text{PP}\left({\text{High}}_{n}\right)}{\frac{1}{N_{\text{Low}}}\sum_{n=1}^{n=N_{\text{Low}}}\text{RMS}\left({\text{Low}}_{n}\right)}\right]$$

### Phonetic and phonological transcription

The recorded audio signals were used for manual and semiautomated transcription of spoken syllables, lexical tones, and corresponding Chinese characters; verification of the participant’s pronunciation accuracy during the tasks; and precise determination of speech onset times to segment the neural data. Acoustic features, including sound intensity and pitch contours (F0), were extracted from the participant’s audio recordings using custom Python scripts. Syllable onsets were identified as the time points when the speech intensity exceeded a manually calibrated threshold, whereas tone onsets were marked at the first appearance of a stable pitch contour. Simultaneously, each Chinese character was automatically transcribed into its corresponding pinyin syllable and tone using custom Python scripts. These annotations were used to generate an initial TextGrid file. Subsequently, all automatic annotations were manually reviewed and refined in Praat (version 6.1.01, https://fon.hum.uva.nl/praat/). Onset times were corrected where necessary, mispronounced syllables were corrected according to participant’s actual pronunciation, and trials that contain significant disfluencies or acoustic artifacts were excluded.

### t-SNE visualization for neural representation analysis

To qualitatively assess the stability of neural representations across recording days, we visualized HGA epochs using t-SNE. For this analysis, HGA epochs from each character reading, spanning ±1.2 s relative to speech onset including all channels, were first reshaped into single vectors. These vectors were then standardized (*z*-scored using sklearn.preprocessing.StandardScaler). Subsequently, t-SNE (sklearn.manifold.TSNE) was implemented using its default hyperparameter settings to generate a two-dimensional (2D) embedding for each epoch. The resulting 2D points were then plotted and colored by recording day for visual inspection of data structure and separability.

### Cortical surface and electrode reconstruction

For precise electrode localization, a Medtronic neuronavigation system was used during the surgical procedure to register the 3D spatial coordinates of the ECoG grid’s corner points. These registered corner coordinates were then carefully aligned with the participant’s preimplantation MRI data, referencing intraoperative images to ensure accuracy. The Python-based “img_pipe” toolkit (*41*) was used to reconstruct the brain surface, generate visualizations of the electrode array on the basis of established corner coordinates and the array’s intrinsic physical geometry, and assign anatomical labels to individual electrodes.

### Speech-responsive electrodes

To identify electrodes responsive to speech production, HGA was analyzed from all syllable-reading trials. For each trial, an “onset period” was defined as the 1000-ms window of HGA data centered on the acoustically determined syllable onset (−500 to +500 ms relative to onset). A “baseline period” of 200 ms was defined for each trial, commencing before onset (−700 to −500 ms relative to onset). To ensure balanced sample sizes for statistical comparison, 1000 “onset period” segments and 1000 corresponding “baseline period” segments were randomly selected from the total trials. For statistical analysis, a single mean baseline value was first calculated for each trial by averaging the HGA across its “baseline period.” Then, at each time point within the “onset period,” a paired *t* test (scipy.stats.ttest_rel) was conducted to compare the instantaneous HGA value against the mean baseline from that same trial. Electrodes were identified as speech-responsive if they exhibited statistically significant differences (*P* < 0.05, Bonferroni corrected for the total number of electrodes and time points) for a continuous duration of at least 200 ms.

### Syllable and tone discriminative electrodes

To identify electrodes that robustly discriminate between syllables, we segmented the HGA for each trial into windows spanning from 1.2 s before to 1.2 s after syllable onset. A spatiotemporal cluster-based permutation test was used to detect time-electrode clusters showing significant effects across syllable conditions. At each time point on each electrode, a one-way ANOVA was conducted to compute the *F*-statistic. To account for electrode proximity, an adjacency matrix based on interelectrode distance was constructed. Cluster-level permutation testing was performed with 1000 permutations using the mne.stats.spatio_temporal_cluster_test function (MNE-Python), an *F*-threshold of 3.0, and a cluster-level alpha of 0.05. From the resulting significant clusters, we further identified syllable-discriminative electrodes by conducting pairwise Welch’s *t* tests (scipy.stats.ttest_ind) across all syllable pairs. An electrode was considered discriminative if it significantly distinguished (*P* < 0.05, false discovery rate corrected for all multiple comparisons) at least three different syllables over a continuous window of 200 ms (≥80 consecutive time points).

A distinct statistical procedure was used to identify electrodes that discriminate lexical tones. To ensure balanced sample sizes for statistical comparison, 1000 segments for each tone were randomly selected from the total trials. For each electrode and each time point within the trial window, a one-way ANOVA (scipy.stats.f_oneway) was conducted to compare high-γ activity across the four Mandarin tone conditions. An electrode was lastly defined as tone discriminative if it exhibited a continuous period of significant activity (*P* < 0.05, Bonferroni corrected for the total number of electrodes and time points) for at least 200 ms (≥80 consecutive time points).

### Articulation manner and place discriminative electrodes

To identify electrodes encoding specific articulatory features of initial consonants in Mandarin Chinese syllables, we categorized initials on the basis of their manner of articulation and, separately, their place of articulation. Initial consonants were grouped into five categories on the basis of their manner of articulation (Plosive, Fricative, Affricate, Nasal, and Lateral). To identify electrodes that significantly differentiated among these multiple (≥3) manner categories, we used the same statistical methodology previously described for identifying syllable-discriminative electrodes. For place of articulation, initial consonants were categorized into two primary groups (Labial versus Lingual). To identify electrodes sensitive to this feature, a paired *t* test was performed for each electrode at each time point. An electrode was considered discriminative to place of articulation if it showed a significant difference (*P* < 0.05) for a continuous period of at least 200 ms (≥80 consecutive time points).

### Speech onset detection

For audio-based onset detection, the real-time audio signal was first normalized to the range of [−1, 1]. Short-time power was then computed using a sliding window of 25 ms. To reduce noise and enhance stability, the power signal was smoothed using a moving average filter. Speech onset was subsequently identified by applying a threshold set at 1.6 times the mean of the smoothed power signal.

For neural-based onset detection, a speech onset detection module, informed by a prior work (*40*), was implemented to identify speech initiation from continuous 100-Hz HGA features (fig. S7). For offline model training, a 15–time point (150 ms) bidirectional contextual HGA feature vector (current time *t* ± 7 time points) was constructed for each time point *t* and labeled as “onset” or “non-onset” using ground-truth annotations. These high-dimensional contextual features were reduced to their top 50 principal components via principal components analysis. These components then trained a Linear Discriminant Analysis (LDA) classifier to output a continuous speech onset probability. This probability was smoothed (2–time point/20-ms window), and an onset was declared if the smoothed probability exceeded 0.45 for 14 consecutive windows (140 ms). For real-time decoding, the onset detection module used the same bidirectional 150-ms feature window (current time *t* ± 70 ms) as in offline training (resulting in a fixed 210-ms detection latency). All critical detection parameters were optimized via random search on a development dataset to maximize the *F*1 score.

### Syllable decoder architecture and training

The primary syllable decoder aimed to classify neural feature segments into specific Mandarin Chinese syllables. Input to the decoder consisted of 1000-ms segments of preprocessed neural features, centered around the detected speech onset (from −300 to +700 ms relative to onset). We evaluated several architectures, including a CNN-LSTM model (architecture and parameters detailed in fig. S8A and table S3), a ViT model (details in fig. S8B and table S4) (*42*), and a four-layer stacked LSTM network (termed “Stacked LSTM”; see Fig. 3A and table S5 for details). The ViT model adapted the standard architecture for 2D image patches by converting the input neural signal (time × channels) into a 2D-like matrix, segmenting it into overlapping patches, flattening and linearly projecting these patches into embedding vectors, prepending a CLS (classification) token, and processing the sequence through standard Transformer encoder blocks; the CLS token’s output representation was then used for classification. The Stacked LSTM architecture comprised two core blocks, each containing a two-layer bidirectional LSTM followed by Layer Normalization and Dropout. The first block transformed 250-dimensional input features to 500 dimensions, and the second processed these into 200-dimensional features, which were then passed to a fully connected layer for mapping to syllable probability distributions. Final syllable probabilities were obtained by averaging the per-timestep predictions across the entire input. All models were trained using the Adam optimizer, ReduceLROnPlateau learning rate scheduler, and a mixup data augmentation. Specific hyperparameters for each model were optimized as detailed in their respective supplementary tables.

### Tone decoder architecture and training

A dedicated tone decoder was developed to identify the lexical tone of each spoken syllable. This decoder used a similar architectural design and training methodology as the selected syllable decoder (see Fig. 3A for details). The input neural features for tone decoding were identical to those used for syllable decoding (1000-ms segments time-locked to speech onset), and the output layer was designed to classify among the Mandarin tone categories. Because the four tones had different proportions in the training set, a focal loss function was used during training. Hyperparameters for the tone decoder were independently optimized to maximize tone classification accuracy.

### Language model

To enhance the naturalness and accuracy of decoded output, particularly at the sentence level, a statistical language model was integrated into the BCI system. A 3-gram model was implemented, combined with a beam search algorithm for sequence selection. This language model was trained on a corpus consisting of the predefined set of sentences used in the experimental tasks. During sentence decoding, outputs from the syllable and tone decoders were first used to generate a list of candidate Chinese characters with associated neural likelihoods, based on a predefined syllable-to-character dictionary. The beam search algorithm then computed the joint probability of the top 10 candidate Chinese character, integrating the neural model likelihoods with the linguistic probabilities provided by the language model, to determine the most probable sequence as the final output.

### Performance evaluation

Single-unit decoding: The performance of the syllable and tone decoders was primarily evaluated offline using data from the single-character reading task, which covered 394 distinct Mandarin tonal syllables (table S1). A 10-fold cross-validation procedure was used. In each fold, 10% of the total dataset (all trials for all 394 syllables) was randomly selected as the test set. The remaining 90% was further partitioned into training (70% of this remainder, 63% of total) and validation sets (30% of this remainder, 27% of total). The primary evaluation metric was classification accuracy.

In addition, we investigated how decoding accuracy was influenced by the number of training repetitions per syllable. Specifically, we selected 5, 10, 15, and 20 repetitions per syllable from the full dataset and performed 10-fold cross-validation for each case. If a syllable had fewer available instances than the target repetition number, all available instances were used. We further examined the impact of the total number of unique syllables in the training set on decoding performance. We randomly selected 50, 100, 150, 200, 250, 300, and 350 syllables from the full set, and decoding was performed for each subset. Each syllable count condition was repeated 10 times with different random selections to ensure robust evaluation.

Real-time sentence decoding: Sentence decoding performance was assessed using a dedicated dataset of sentences in real-time experimental sessions on the final day. The neural decoders (syllable and tone, fine-tuned on sentence data) and the language model were trained and validated independently. During testing, the probabilistic outputs from the fine-tuned neural decoders were processed by the language model. The primary metric was CAR, defined as the percentage of correctly decoded Chinese characters relative to the total number of characters in the ground truth sentences (correct number of detected/total number). Performance was reported for both neural decoding alone and for the integrated system with the language model. For real-time decoding tasks, the chance level performance was defined as 1 divided by the total number of possible output classes. The overall decoding speed was calculated on the basis of system latency and reported in CPM.

### Electrode contribution analysis

To quantify each ECoG electrode’s contribution to the decoder’s performance, we used a gradient-based input saliency analysis (*43*). This method involved calculating the cross-entropy loss between the model’s predictions and true labels for a given dataset. We then computed the gradients of this loss with respect to the input HGA features. The contribution score for each electrode was derived by first summing the absolute values of these gradients across all time points for each trial and then averaging these sums across all trials. Last, these aggregate scores were normalized to a [0, 1] range, where higher values indicate greater electrode influence on the decoding outcome.

### Statistical analyses

Specific statistical tests and parameters are detailed in the relevant figure captions and main text. In summary, to compare decoding accuracies across multiple independent groups, one-way AANOVA was used, followed by Tukey’s post hoc tests for pairwise comparisons. Before these analyses, data were confirmed to meet assumptions of normality via Shapiro-Wilk tests and homogeneity of variances (via the Brown-Forsythe test). To assess whether decoding accuracies for individual conditions were significantly above chance level, one-sample *t* tests were used, and *P* values were adjusted using the Bonferroni correction to control for the family-wise error rate. Unless otherwise specified, a *P* value of <0.05 was considered statistically significant. All reported 99% CIs were estimated using a bootstrap procedure with 1000 resamples.
