# Supplementary material for: Real-time decoding of full-spectrum Chinese using brain-computer interface
Source: Sci Adv. 2025 Nov 5;11(45):eadz9968. doi: 10.1126/sciadv.adz9968 (PMC12588286; doi:10.1126/sciadv.adz9968)
Supplement: Supplementary file 1 — Figs. S1 to S10 Tables S1 to S5 Legend for data S1 [file sciadv.adz9968_sm.pdf]

Science Advances
AAAS

Supplementary Materials for

Real-time decoding of full-spectrum Chinese using brain-computer interface

Youkun Qian *et al.*

Corresponding author: Jinsong Wu, wujinsong@huashan.org.cn; Zhitao Zhou, ztzhou@mail.sim.ac.cn

Sci. Adv. **11**, eadz9968 (2025)
DOI: 10.1126/sciadv.adz9968

The PDF file includes:

Figs. S1 to S10
Tables S1 to S5
Legend for data S1

Other Supplementary Material for this manuscript includes the following:

Data S1

Fig. S1.

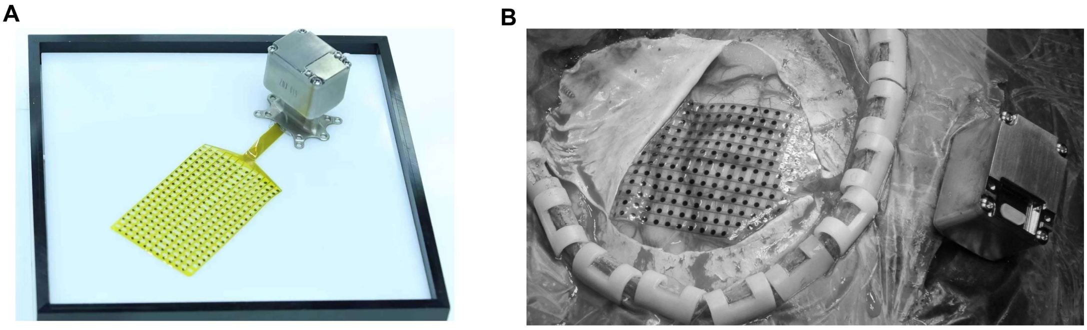

Fig. S1. Intraoperative photographs of ECoG electrode implantation. A. Photograph showing the flexible high-density ECoG grid array and its headstage, which provides amplification and digitization of the neural signals near the source. **B.** Photograph taken during surgical implantation showing the placement of the flexible high-density ECoG grid array on the cortical surface.

Fig. S2.

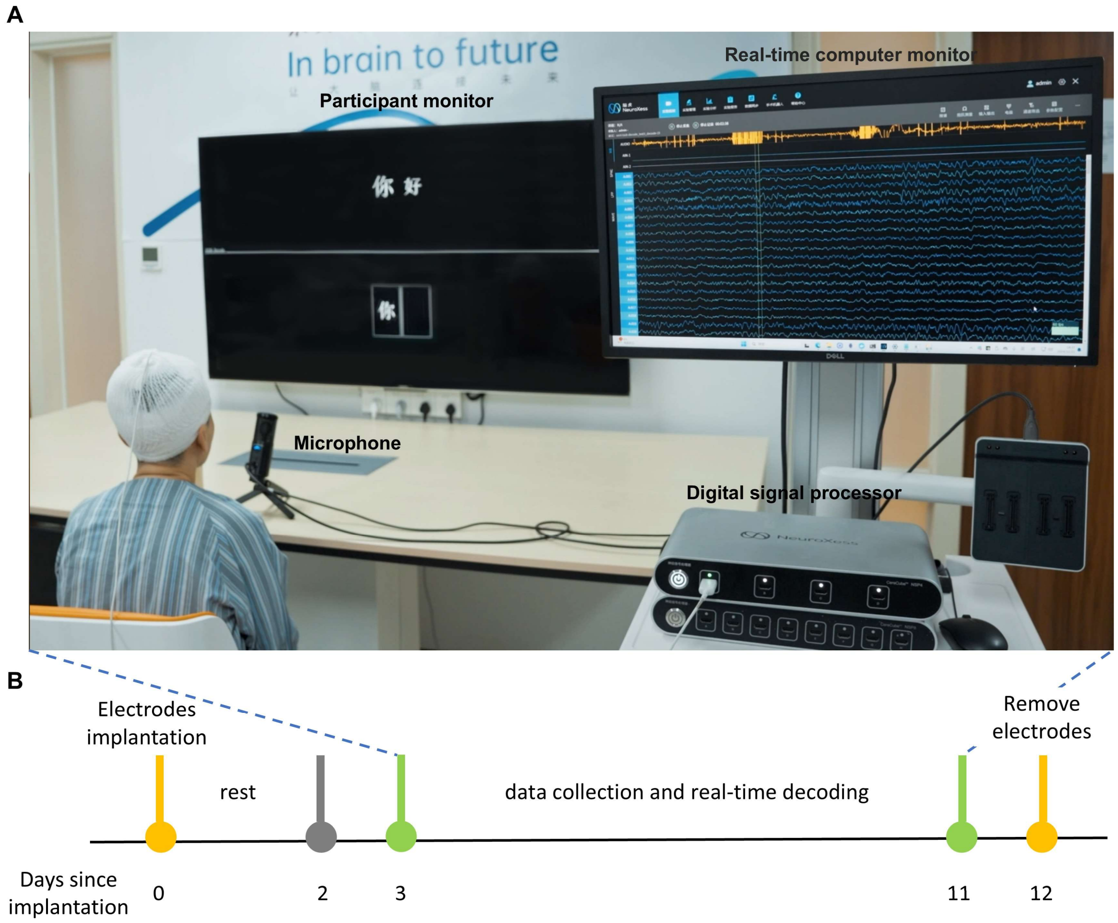

Fig. S2. Experimental Setup and Timeline for Data Acquisition. A. Data Acquisition Environment. The participant was comfortably seated by a table during experimental task performance. Electrocorticography (ECoG) signals were continuously recorded from the implanted electrode array. Simultaneously, a microphone captured the participant's vocal responses as audio signals. Both the ECoG and microphone signals were synchronously transmitted to a digital signal processor for precise temporal alignment and signal amplification before subsequent offline analysis and real-time decoding. **B.** After the surgical implantation of the ECoG array, the participant rest for two days. Experimental sessions start from the third day since implantation, after the participant reported no discomfort and was deemed clinically stable. Data collection continued for nine days. The ECoG electrodes were surgically explanted on the twelfth day since implantation.

Fig. S3.

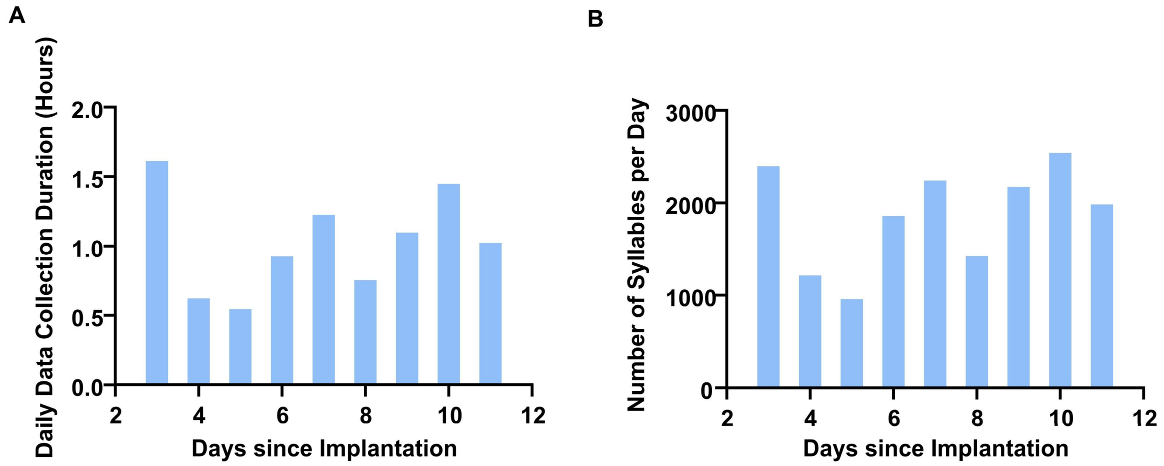

Fig. S3. Daily task completion and duration during the character-reading task. The figure summarizes experimental activity from day 3 to day 11 post-electrode implantation. **A.** Daily duration (in hours) of data collection in character-reading task. **B.** Total number of syllables recorded in character-reading task per day, representing daily task volume.

Fig. S4.

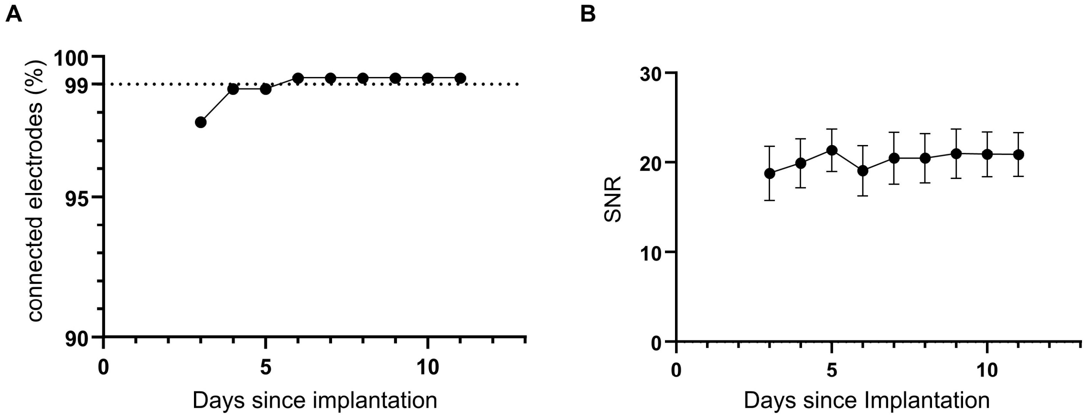

Fig. S4. Longitudinal stability and signal characteristics of implanted ECoG electrodes. The figure illustrates changes in electrode integrity and signal properties over the implantation period. **A.** The number of channels identified as "bad" or "noisy" each day, based on excessive baseline impedance. **B.** The daily average signal-to-noise ratio (SNR) value calculated across all good channels, indicating overall signal amplitude over noise levels. The x-axis represents days since surgical implantation. Error bars represent standard error of the mean across channels.

Fig. S5.

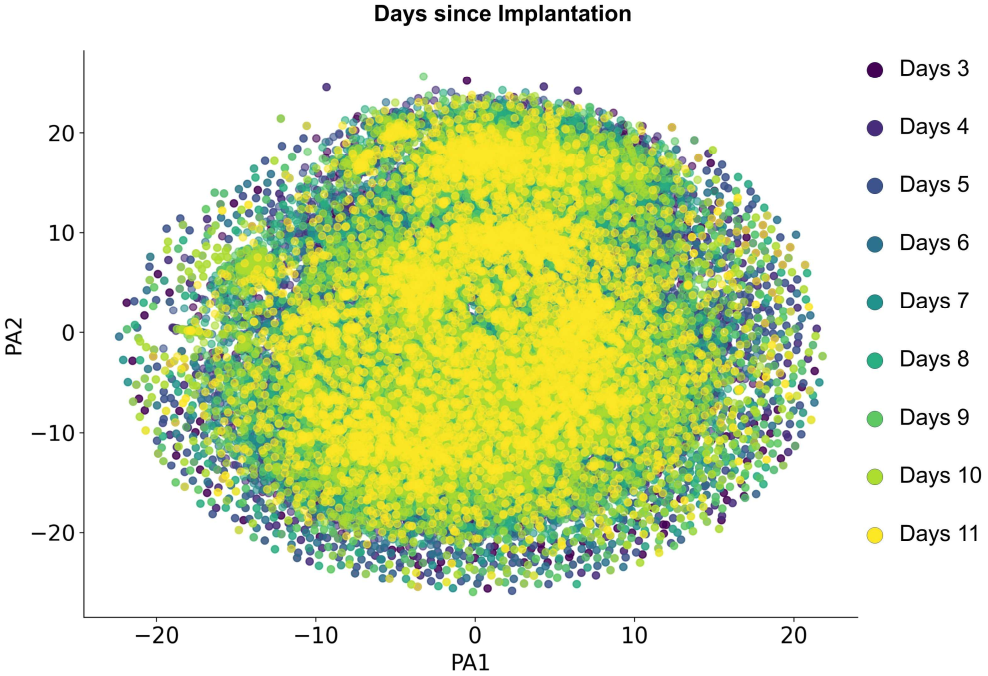

Fig. S5. t-SNE visualization of ECoG data features demonstrating stability across days and task contexts. t-SNE projection of neural features extracted from ECoG data, with individual data points colored by the recording day (days 3-11 post-implantation). The visualization shows substantial overlap between data from different days, suggesting no clear systematic drift or clustering by day.

Fig. S6.

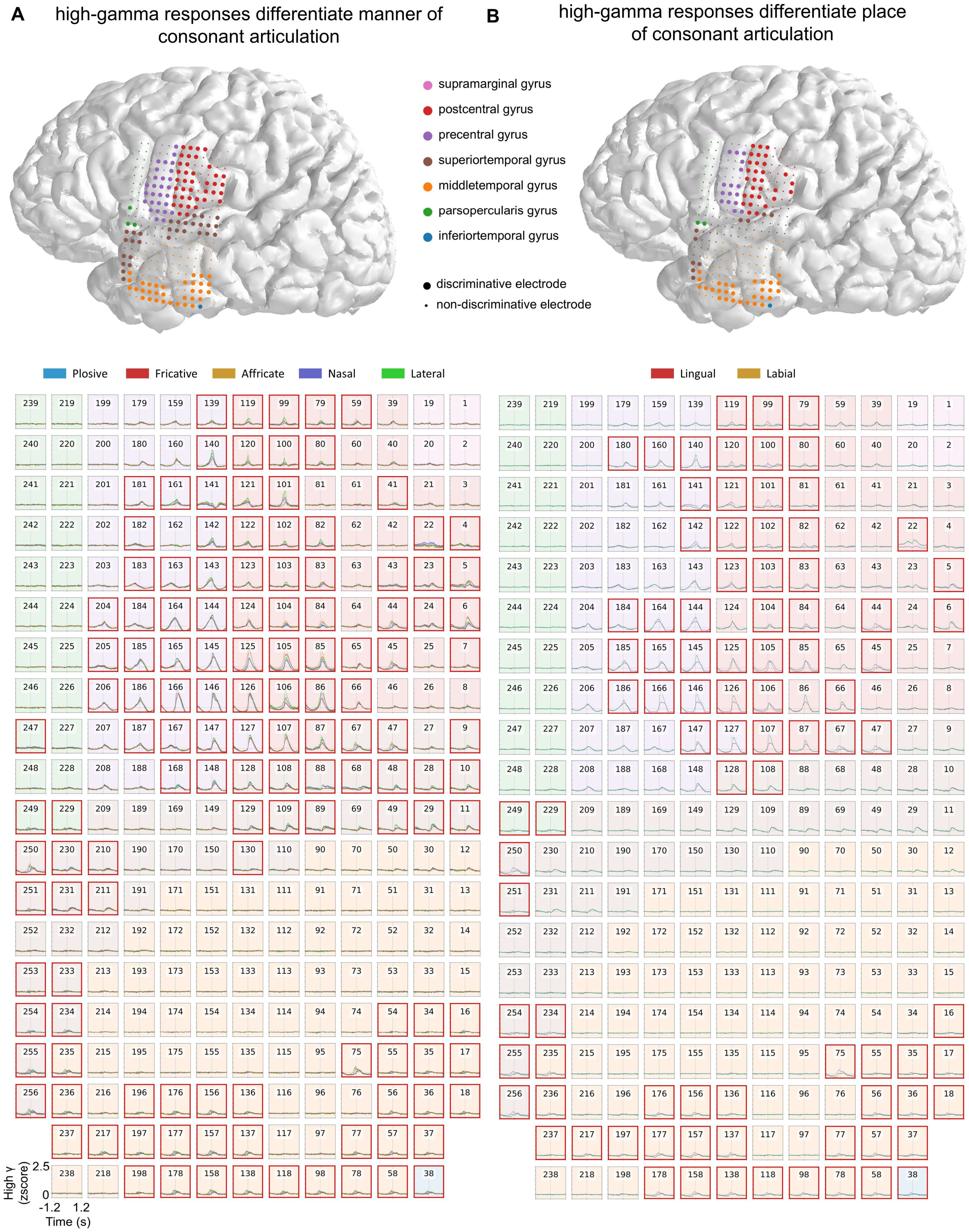

Fig. S6. High-γ activity associated with the manner and place of consonant articulation. Mean high-γ activity (70-150 Hz; ±1200 ms relative to speech onset) is shown for representative ECoG channels, averaged for consonants grouped by (**A**) manner of articulation and (**B**) place of articulation. Channels where high-γ signals significantly differentiated between distinct manner categories or place categories are outlined with red boxes in the respective plots and their locations are projected onto a template brain model ($p < 0.05$, cluster-based permutation test). Manner of articulation categories included:

Plosive, Fricative, Affricate, Nasal, and Lateral. Place of articulation categories included: Lingual and Labial.

Fig. S7.

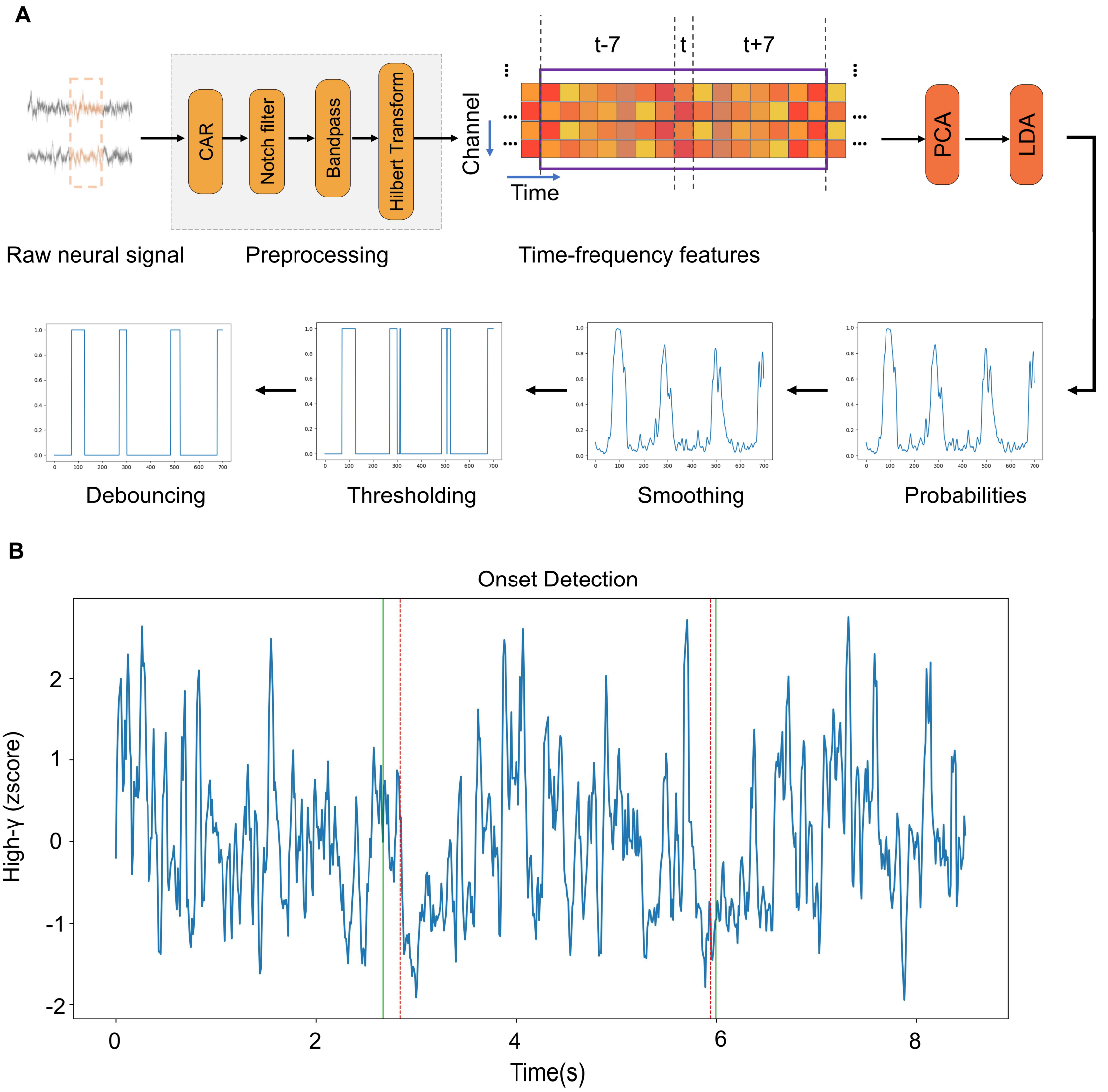

Fig. S7. Speech onset detection architecture and application to covert speech. A. Architecture of the real-time speech onset detector. Following initial data preprocessing, features are extracted using Principal Component Analysis and Linear Discriminant Analysis. Speech probability is then computed, smoothed, and thresholded to identify speech segments. **B.** Successful application of the onset detection algorithm to ECoG data from an independent epilepsy patient engaged in a covert speech task. The continuous high γ signals (blue trace) is plotted over time. The algorithm's predicted onsets (solid green lines) show close alignment with the ground-truth onsets (dashed red lines), which were determined from mouth movements.

Fig. S8.

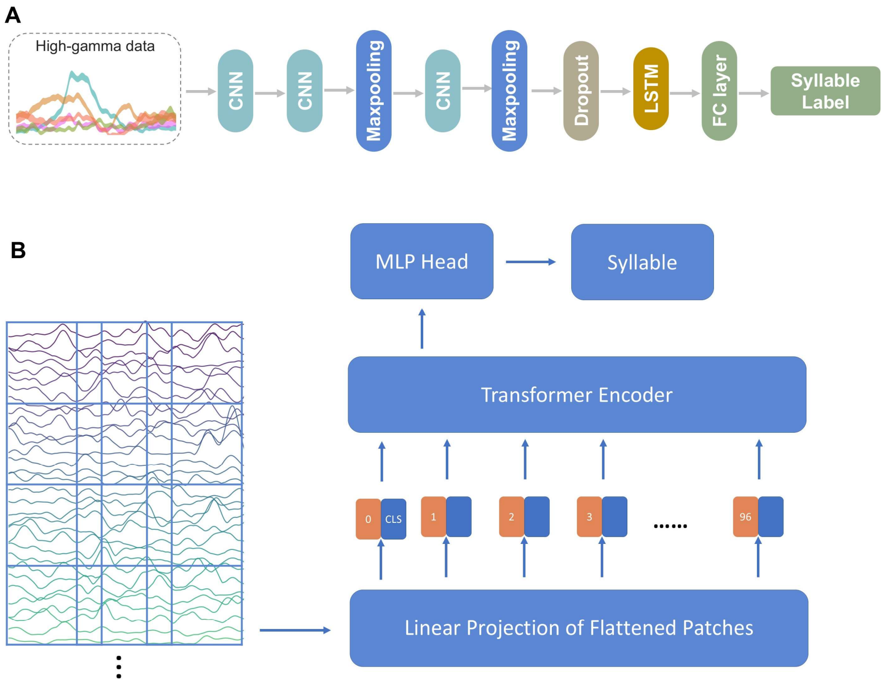

Fig. S8. CNN-LSTM and Vision Transformer (ViT) architecture for syllable decoding. A. The CNN-LSTM model, which consists of three convolutional layers followed by a LSTM layer to decode syllables. Detailed hyperparameters are in table S3. **B.** The ViT architecture. Neural segment is treated as a 2D representations (channels × time) and divided into overlapping patches. These patches, combined with positional embeddings and a classification token, are then processed by a transformer encoder and MLP head for classification. Detailed hyperparameters are provided in table S4.

Fig. S9.

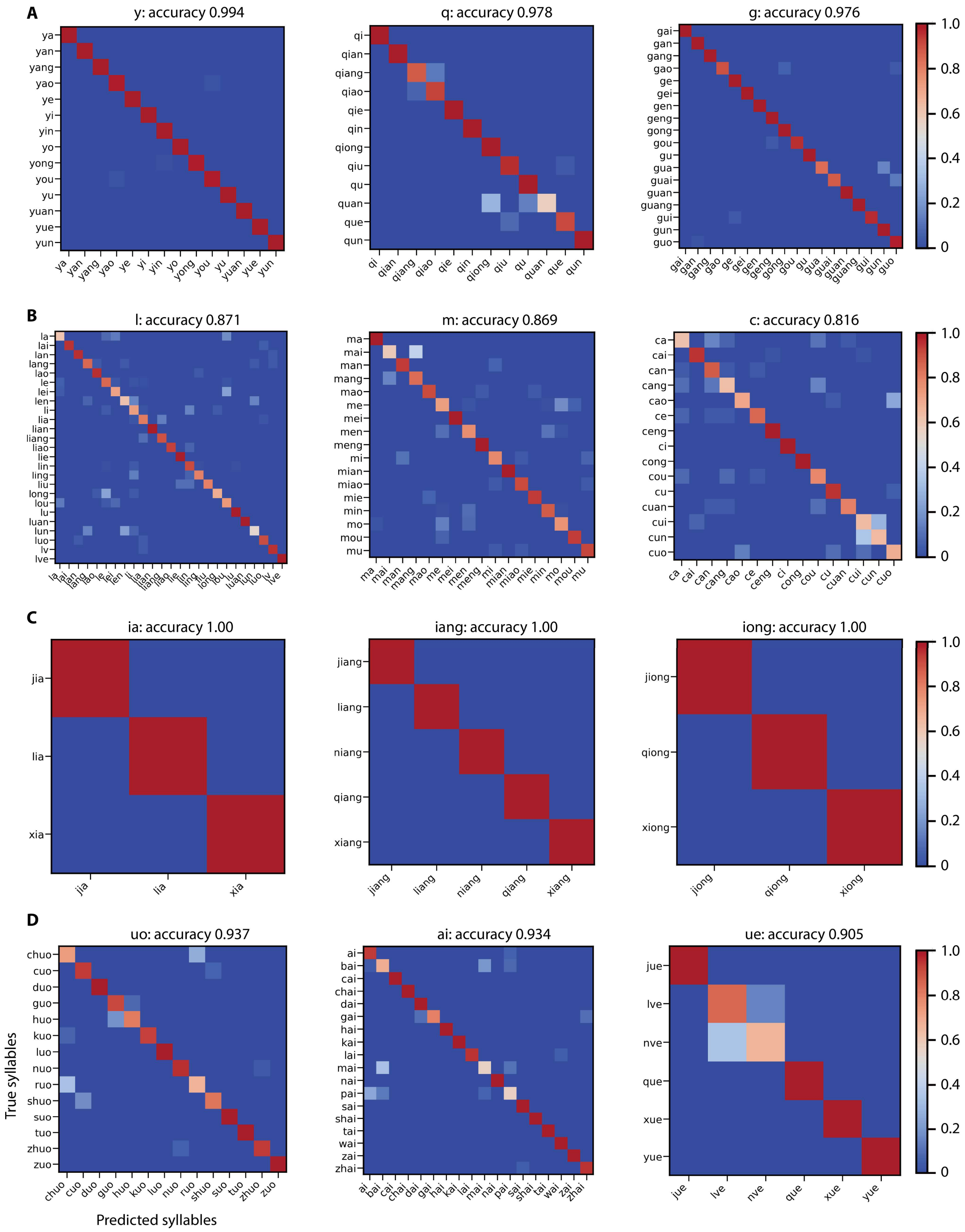

Fig. S9. Initial and final subgroup confusion matrix. Decoding accuracy for each initial or final is calculated internally within each subgroup. **A.** Three initial-based subgroups with the highest internal accuracy (y /j/, q /tɕʰ/, g /k/) **B.** Three initial-based subgroups with the with the lowest internal accuracy (l /l/, m /m/, c /tsʰ/). **C.** Three finals with the best internal accuracy (ia [ja], iang [jɑŋ], iong [jʊŋ]) **D.** Three finals with the lowest internal accuracy

(uo [wo], ai [ai̯], ue [ɥe]). Color bar represents the prediction probability for each true syllable.

Fig. S10.

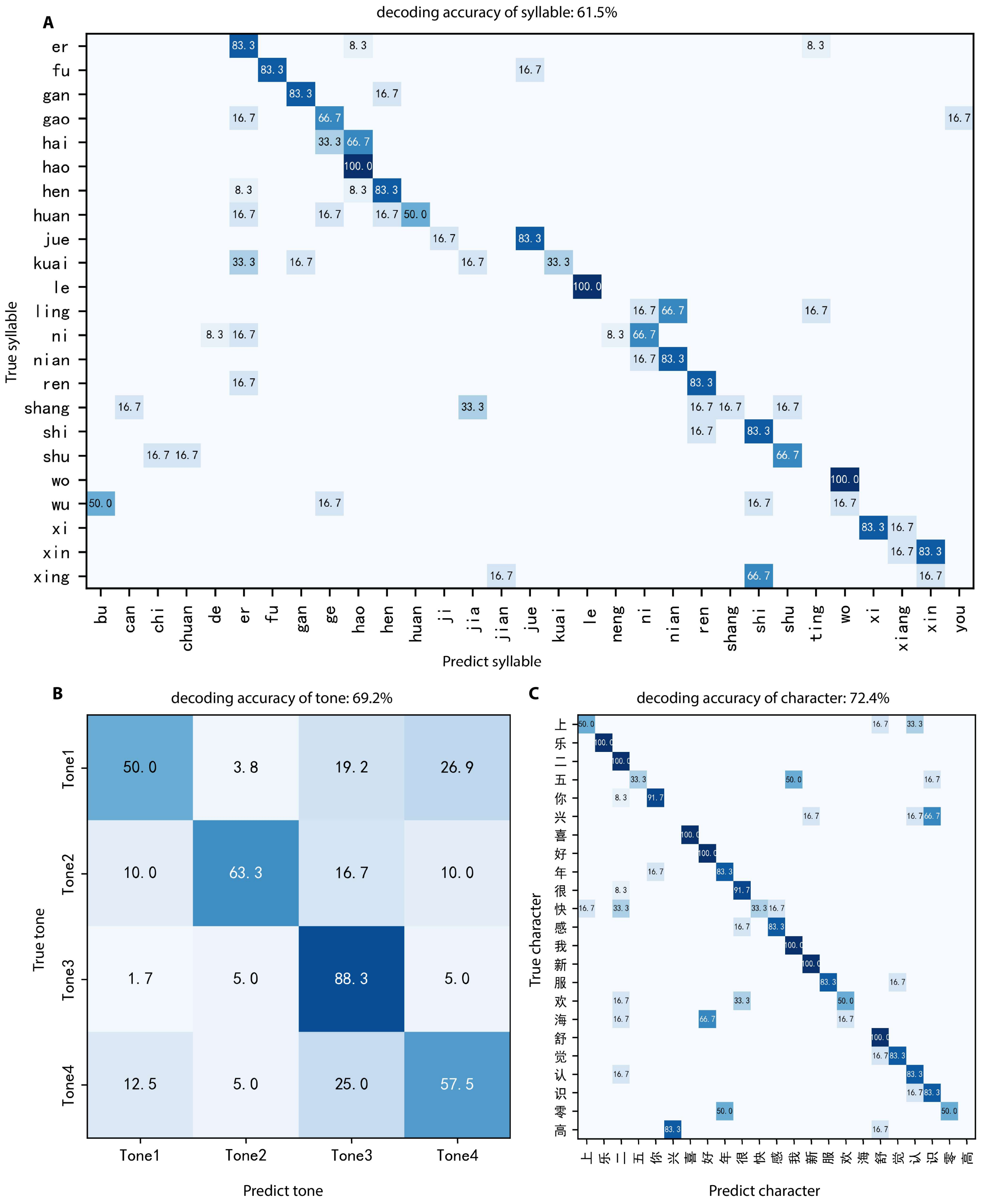

Fig. S10. Syllable, tone and character confusion matrix in real-time sentence decoding task. (A) Confusion matrix for syllable decoding without the language model, showing a mean accuracy of 61.5%. **(B)** Confusion matrix for tone decoding, with a mean accuracy of 69.2%. **(C)** Confusion matrix for the final character output with the language model, achieving a mean accuracy of 72.4%. Given that the sentence stimuli did not include characters differing only in tone, all tonal variants of a syllable were mapped to a single representative character during real-time decoding.

Table S1.

Final

Initial	Simple finals						Compound finals									Anterior nasal finals				
	a	o	e	i	u	ü	ai	ei	ui	ao	ou	iu	ie	üe	er	an	en	in	un	ün
*	a	o	e				ai	ei		ao	ou				er	an	en			
b	ba	bo		bi	bu		bai	bei		bao			bie			ban	ben	bin		
p	pa	po		pi	pu		pai	pei		pao	pou		pie			pan	pen	pin		
m	ma	mo	me	mi	mu		mai	mei		mao	mou	miu	mie			man	men	min		
f	fa	fo			fu			fei			fou					fan	fen			
d	da		de	di	du		dai	dei	dui	dao	dou	diu	die			dan	den		dun	
t	ta		te	ti	tu		tai	tei	tui	tao	tou		tie			tan			tun	
n	na		ne	ni	nu	nü	nai	nei		nao	nou	niu	nie	nüe		nan	nen	nin	nun	
l	la	lo	le	li	lu	lü	lai	lei		lao	lou	liu	lie	lüe		lan		lin	lun	
g	ga		ge		gu		gai	gei	gui	gao	gou					gan	gen		gun	
k	ka		ke		ku		kai	kei	kui	kao	kou					kan	ken		kun	
h	ha		he		hu		hai	hei	hui	hao	hou					han	hen		hun	
j				ji		ju						jiu	jie	jue				jin		jun
q				qi		qu						qiu	qie	que				qin		qun
x				xi		xu						xiu	xie	xue				xin		xun
zh	zha		zhe	zhi	zhu		zhai	zhei	zhui	zhao	zhou					zhan	zhen		zhun	
ch	cha		che	chi	chu		chai		chui	chao	chou					chan	chen		chun	
sh	sha		she	shi	shu		shai	shei	shui	shao	shou					shan	shen		shun	
r			re	ri	ru				rui	rao	rou					ran	ren		run	
z	za		ze	zi	zu		zai	zei	zui	zao	zou					zan	zen		zun	
c	ca		ce	ci	cu		cai	cei	cui	cao	cou					can	cen		cun	
s	sa		se	si	su		sai	sei	sui	sao	sou					san	sen		sun	
y	ya	yo	ye	yi		yu				yao	you			yue		yan		yin		yun
w	wa	wo			wu		wai	wei								wan	wen			
	Two-part syllables																			

Initial	Posterior nasal finals				compound finals that include a medial glide										
	ang	eng	ing	ong	i-a	i-an	i-ang	i-ao	i-ong	u-a	u-ai	u-an	u-ang	u-o	ü-an
*	ang	eng													
b	bang	beng	bing			bian		biao							
p	pang	peng	ping			pian		piao							
m	mang	meng	ming			mian		miao							
f	fang	feng													
d	dang	deng	ding	dong	dia	dian		diao				duan		duo	
t	tang	teng	ting	tong		tian		tiao				tuan		tuo	
n	nang	neng	ning	nong	nia	nian	niang	niao				nuan		nuo	
l	lang	len	ling	long	lia	lian	liang	liao				luan		luo	
g	gang	geng		gong						gua	guai	guan	guang	guo	
k	kang	keng	king	kong						kua	kuai	kuan	kuang	kuo	
h	hang	heng		hong						hua	huai	huan	huang	huo	
j			jing		jia	jian	jiang	jiao	jiong						juan
q			qing		qia	qian	qiang	qiao	qiong						quan
x			xing		xia	xian	xiang	xiao	xiong						xuan
zh	zhang	zheng		zhong						zhua	zhuai	zhuan	zhuang	zhuo	
ch	chang	cheng		chong						chua	chuai	chuan	chuang	chuo	
sh	shang	sheng								shua	shuai	shuan	shuang	shuo	
r	rang	reng		rong						rua		ruan		ruo	
z	zang	zeng		zong								zuan		zuo	
c	cang	ceng		cong								cuan		cuo	
s	sang	seng		song								suan		suo	
y	yang		ying	yong											yuan
w	wang	weng													
	Two-part syllables				Three-part syllables										

special syllable: n | ng, hng

Table S1. Syllable Coverage and Customization for Mandarin Chinese BCI Training. The table displays a comprehensive list of Mandarin Chinese syllables, with color-coding to denote their inclusion and status in this study. Syllables highlighted in green were part of the core training and decoding set. Yellow-highlighted syllables ("king" and "len") represent non-standard but participant-specific pronunciations included for personalized model training. Syllables marked in blue were standard Mandarin syllables excluded from the study due to factors such as participant unfamiliarity, low frequency of use, or time constraints during recording sessions. The final stimulus set consisted of 394 syllables.

Table S2.

Chinese syllable structure

V	VN	CV	CVN

English syllable structure

CV	CVC	CVCC	CVCCC	CVCCCC
CCV	CCVC	CCVCC	CCVCCC	CCVCCCC
CCCV	CCCVC	CCCVCC	CCCVCCC	CCCVCCCC
VC	VCC	VCCC	VCCC	V

Table S2. Linguistic comparison of syllable structures. Comparative overview of syllable structure patterns between Mandarin Chinese and English. This panel illustrates the greater phonotactic complexity allowed in English syllable formation (e.g., consonant clusters) compared to the more constrained structures in Mandarin. (C = consonant, V = vowel).

Table S3.

**Hyperparameter description**	**Final value**
Convolution kernel size	(1,3)
Filter number per CNN layer	64
Maxpooling size1	(1,5)
Maxpooling size2	(1,4)
Hidden units of the LSTM layer	500
Learning Rate	1e-4
Optimizer	Adam
scheduler	ReduceLR On Plateau
Dropout	0.5
Batch size	32
Early-stop patience	30

Table S3. CNN-LSTM neural-decoding model hyperparameter values.

Table S4.

Hyperparameter description	Final value
Patch size	40
Patch channels	8
Transformer depth	8
Embedding dimension	256
Number of heads	16
MLP ratio	8
QKV bias	True
Learning rate	1e-4
Weight decay	1e-5
Optimizer	Adam
Scheduler	Reduce LR On Plateau
Dropout	0.1
Attention dropout	0.1
Batch size	8
Early-stop patience	150

Table S4: Vision Transformer (ViT) neural-decoding model hyperparameter values.

Table S5.

Hyperparameter description	Final value
Number of LSTM layers	4
Hidden units of 1st LSTM layer	250
Hidden units of 2st LSTM layer	250
Hidden units of 3st LSTM layer	100
Hidden units of 4st LSTM layer	100
Learning Rate	8e-4
Weight decay	1e-3
Optimizer	Adam
Scheduler	Reduce LR On Plateau
Dropout	0.1
Batch size	16
Early-stop patience	30

Table S5. 4-layer Stacked LSTM syllable-decoding model hyperparameter values

Data S1.

Data S1. Syllable Coverage and Customization for Mandarin Chinese BCI Training (Excel Format). This file provides the data from Table S1 in an Excel format for improved readability and accessibility, as the original table is densely formatted.
